# A Systematic Review of Information and Communication Technology–Based Interventions for Promoting Physical Activity Behavior Change in Children and Adolescents

**DOI:** 10.2196/jmir.1533

**Published:** 2011-07-13

**Authors:** Patrick WC Lau, Erica Y Lau, Del P Wong, Lynda Ransdell

**Affiliations:** ^1^Department of Physical EducationHong Kong Baptist UniversityHong KongChina; ^2^Department of Health and Physical EducationHong Kong Institute of EducationHong KongChina; ^3^Department of KinesiologyCollege of EducationBoise State UniversityBoiseUnited States

**Keywords:** Internet, email, text messages

## Abstract

**Background:**

A growing body of research has employed information and communication technologies (ICTs) such as the Internet and mobile phones for disseminating physical activity (PA) interventions with young populations. Although several systematic reviews have documented the effects of ICT-based interventions on PA behavior, very few have focused on children and adolescents specifically.

**Objectives:**

The present review aimed to systematically evaluate the efficacy and methodological quality of ICT-based PA interventions for children and adolescents based on evidence from randomized controlled trials.

**Methods:**

Electronic databases Medline, PsycInfo, CINAHL, and Web of Science were searched to retrieve English language articles published in international academic peer-reviewed journals from January 1, 1997, through December 31, 2009. Included were articles that provided descriptions of interventions designed to improve PA-related cognitive, psychosocial, and behavioral outcomes and that used randomized controlled trial design, included only children (6-12 years old) and adolescents (13-18 years old) in both intervention and control groups, and employed Internet, email, and/or short message services (SMS, also known as *text messaging*) as one or more major or assistive modes to deliver the intervention.

**Results:**

In total, 9 studies were analyzed in the present review. All studies were published after 2000 and conducted in Western countries. Of the 9 studies, 7 demonstrated positive and significant within-group differences in at least one psychosocial or behavioral PA outcome. In all, 3 studies reported positive and significant between-group differences favoring the ICT group. When between-group differences were compared across studies, effect sizes were small in 6 studies and large in 3 studies. With respect to methodological quality, 7 of the 9 studies had good methodological quality. Failure to report allocation concealment, blinding to outcome assessment, and lack of long-term follow-up were the criteria met by the fewest studies. In addition, 5 studies measured the intervention exposure rate and only 1 study employed objective measures to record data.

**Conclusion:**

The present review provides evidence supporting the positive effects of ICTs in PA interventions for children and adolescents, especially when used with other delivery approaches (ie, face-to-face). Because ICT delivery approaches are often mixed with other approaches and these studies sometimes lack a comparable control group, additional research is needed to establish the true independent effects of ICT as an intervention delivery mode. Although two-thirds of the studies demonstrated satisfactory methodological quality, several quality criteria should be considered in future studies: clear descriptions of allocation concealment and blinding of outcome assessment, extension of intervention duration, and employment of objective measures in intervention exposure rate. Due to the small number of studies that met inclusion criteria and the lack of consistent evidence, researchers should be cautious when interpreting the findings of the present review.

## Introduction

Regular physical activity (PA) is associated with reduced risk of breast cancer, hypertension, coronary heart disease, type 2 diabetes mellitus, obesity, and osteoporosis in children and adolescents [1-3]. However, the majority of our young population is not engaging in sufficient PA to achieve these health benefits [4-7]. Developing effective interventions to promote active lifestyles among children and adolescents is one way to address the lack of PA in this population.

In the past decade, numerous PA interventions have been developed and implemented [8,9]. The typical modes of delivery for these interventions have been face-to-face and mass media [10,11]. Studies [12-14] have indicated that interventions delivered using a face-to-face approach (ie, structured PA programs and individual counseling) have been effective for PA behavior changes, but effects have been small. Due to time schedules, high running costs, and geographic restrictions, these interventions could not reach and be accessed by a large population [15-18]. With the combination of small effects and limited reach, the impact of face-to-face PA interventions on public health has been modest [19]. On the other hand, PA interventions disseminated through mass media approaches (ie, TV, radio, and pamphlets) have the potential to reach large numbers of individuals. One limitation of mass media–based PA interventions is that they mainly contain generic content and feedback that is less relevant to individuals who may need different strategies to change PA behavior [10]. The aforementioned limitation probably explains why the majority of these mass media–based interventions (except the VERB campaign [20]) were only successful in raising awareness and increasing knowledge and not in improving PA behavioral outcomes (ie, increasing PA levels) [21]. In sum, innovative approaches that can reach large groups of people while at the same time enhancing accessibility and personal relevance are needed. Interestingly, studies have shown that this lack of personalization, mentioned as a limitation of media-based PA interventions, could be addressed with the use of advanced information and communication technology (ICT) such as Internet, personal digital assistants, computer kiosks, and mobile phones [22,23].

### Advantages of ICT-based interventions

The proliferation of the Internet and mobile phones has provided a powerful channel to widen the reach of PA interventions in children and adolescents [23]. In developed countries, over 90% of children and adolescents were found to have had access to the Internet at school and/or home [14]. More importantly, they perceived the Internet as their primary resource for seeking health information [24,25]. Additionally, 45% to 99% of children and adolescents have been found to own a personal mobile phone, and half of them use short message services (SMS, also known as *text messaging*) [10]. In addition to this broadened reach, advanced Web technologies can enhance the personal relevance of an intervention's contents. Researchers can now tailor PA interventions based on a variety of factors that influence PA behavior change in children and adolescents (eg, gender, ethnicity, weight status, stage of change, PA self-efficacy, and PA barriers). In addition, ICT interventions can present materials in various forms (ie, text, sound, video, and animation) to satisfy children’s and adolescents’ preferences [22]. Moreover, email and SMS have provided a means for researchers to deliver individualized feedback, automatic reminders, and social support. These elements could enhance children’s and adolescents’ attention toward and understanding of the materials [26-28], which could lead to subsequent improvements in PA behavior.

In recent years, the evidence base of ICT-based health behavior change interventions has been growing [29-31]. Several systematic reviews [26,28,32-36] have evaluated the efficacy of these interventions; however, very few studies have focused on PA behavior. Even though some studies have concentrated specifically on PA behavior [32,33,35,36], none has focused on children and adolescents. To our knowledge, the systematic review conducted by Norman et al [33] was the only paper published in the previous 5 years that has documented the efficacy of ICT-based PA interventions in children and adolescents. These authors conducted an electronic database search through the year 2005, and they identified 33 studies with PA as an outcome. As ICT-based PA interventions were still in a development stage at that time, studies that focused on children and adolescents were scant. These authors located 5 studies that focused on children and adolescents, and only 3 of them were randomized controlled trials (RCT). Although Norman and colleagues concluded that ICT-based interventions were effective for changing PA behavior, this conclusion was mainly drawn using evidence collected with adult subjects. Whether their conclusions can be generalized to children and adolescents needs further investigation. Since 2005, several additional RCTs have been published. It is, therefore, timely to conduct a new systematic review to evaluate the efficacy and methodological quality of ICT-based interventions relative to promoting PA behavior change in children and adolescents.

Although there are various ICTs, the present review focused on the Internet and SMS only. The reason for limiting the scope of ICT to Internet, email, and SMS is that these modes are most frequently used among children and adolescents. Other ICTs such as interactive CD-ROMs and computer kiosks were excluded because they are less popular, and, in that format, the advantages of ICT-based interventions (eg, free of time and geographic restrictions) are not fully utilized. Moreover, ICT-based PA interventions for children and adolescents are still in a developing stage; a systematic review focused on both Internet and SMS should demonstrate their usefulness in various research designs. This could inform the choice of ICT in future studies. 

The purpose of the present review was to systematically evaluate the efficacy and methodological quality of ICT-based interventions that applied Internet and/or SMS as a delivery mode for PA behavior change in children and adolescents based on evidence from randomized controlled trials.

### Definitions

In this review, an ICT-based intervention is defined as an intervention that employs Internet, email, and/or SMS as one of the intervention delivery modes. The following types of intervention are excluded from this definition: (1) interventions that only involved ICT for data collection (ie, online surveys and electronic medical records) and (2) interventions that used a computer to generate individually tailored printed materials and delivered those materials using a non-ICT mode. The aforementioned interventions were excluded because there was little or no information exchanged, and interactions between the ICTs and participants were minimal. 

## Methods

An electronic database search was conducted to retrieve English articles from CINAHL, Medline, PsycInfo, PubMed, and Web of Science. The search targeted articles published from January 1, 1997, through December 31, 2009, because ICT-based interventions began in the late 1990s [11]. For CINAHL, Medline, PsycInfo, and Web of Science, we performed a keyword search using the following search strings: (child* OR adolescent* OR teenag* OR youth) AND (Internet OR Web-based OR Web-delivered messages OR email OR e-mail OR electronic mail OR mobile phone OR text messag* OR SMS) AND (daily physical activit* OR exercise OR walk* OR motor activ* OR leisure activit* OR physical fitness OR sport*) AND (health OR health behavior OR weight loss OR obesity OR overweight). In addition, we conducted a MeSH search in PubMed using the following search strings: ("Adolescent"[Mesh] OR "Child"[Mesh]) AND ("Internet"[Mesh] OR "Telecommunications"[Mesh]) AND ("Exercise"[Mesh] OR "Motor Activity"[Mesh] OR "Sports"[Mesh]) AND ("Health Behavior"[Mesh] OR "Obesity"[Mesh] OR "Weight Loss"[Mesh]). 

### Selection Criteria 

To be included, articles had to (1) be published in international academic peer-reviewed journals (book chapters, abstracts of conference proceeding, and dissertations were excluded); (2) use a randomized controlled trials design; (3) evaluate an intervention that aimed to promote PA behavior; (4) include at least one PA behavior variable as the outcome (no restriction was defined regarding the types of PA behavior outcomes, which could be cognitive [ie, PA knowledge], psychosocial [eg, PA intention, PA self-efficacy, social support to PA, stage of change], or behavioral [ie, energy expenditure, step counts, or self-reported PA level]; (5) focus only on children (6-12 years old) and adolescents (13-18 years old) in both the intervention and control group; and (6) employ Internet, email, and/or SMS as one or more major or assistive modes to deliver the intervention. No further limits were set on the types and content of the control group. Control groups were non–ICT-based, no treatment, or different types of ICT-based interventions. 

To attain additional eligible articles, the reference list of the located studies and relevant reviews were also checked. The selection of articles was independently performed by two investigators (authors PWCL and EYL).

### Data Extraction

The present review provided a narrative evaluation of the selected articles because of the heterogeneity in study designs, measures, and outcomes across studies. Information about the selected articles was extracted into a structured summary table by one investigator (EYL) and checked by another investigator (PWCL). The following data were extracted: source (year of publication, country in which study was conducted); study characteristics (study design, setting that the information was delivered via ICT, and target behavior); participant characteristics (sample size, age, and group of participants); intervention descriptions, intervention characteristics (intervention duration, mode of delivery, contact frequency, theoretical basis, types and numbers of behavior change technique [BCT] used, and ICT initiation strategy). Use of BCT was coded according to the definition of the taxonomy of BCT developed by Abraham and Michie [37]. ICT initiation strategy was divided into participant-initiated (participants have to decide when, where, and what information to access or transmit by ICT) or investigator-initiated (an investigator delivered the information to participants via ICT at a fixed time, venue, and under specific conditions). For example, with a participant-initiated process, participants were told to access a PA website twice a week during their free time, but they were not told which day of the week or number of pages to read each time. For an investigator-initiated process, participants might receive a hyperlink via their personal email or individual feedback to their mobile phone.

### The Efficacy of ICT-Based PA Interventions for Children and Adolescents

In addition, types of outcome measures and main findings were coded. The pre-post difference on PA behavior outcome in the intervention group was coded as “↑” for positive and significant change, “→” for no significant change and “↓” for significant negative change. The pre-post difference in PA behavior outcome between the intervention group and control group was coded as “+” (significant difference favoring the ICT intervention group), “O” (no significant difference between groups), and “—” (significant difference favoring the control group). To compare the potential effect of ICT interventions on children and adolescents across studies, an effect size (ES) was calculated when sufficient information was reported. An ES of less than 0.5 was interpreted as small, 0.5 to 0.8, as medium, and greater than 0.8 as large [38]. When a study measured outcomes across several time points, the longest follow-up was selected for effect size calculation. For example, if a study measured exercise behavior at 6, 12, 18, and 24 months the 24-month data were selected for comparison. For studies that employed more than one comparison group, following previous systematic reviews of ICT-based intervention [26,34], the control group with the least contact was selected for ease of interpretation. To ensure the accuracy of the data extraction, original authors of the included studies were contacted for further information and clarification when needed.

### Assessment of Methodological Quality

Methodological quality was assessed using a 13-item scale developed in a previous review [35]. Studies were rated independently by one investigator (EYL) and checked by another investigator (PWCL). Disagreements were discussed until consensus was reached. Each item was rated as *yes*, *no*, or *unknown*. A total methodological quality score (ranging from 0-13) was calculated by summing up all *yes* items. Studies were rated as having good methodological quality if they met at least two-thirds of the criteria (ie, ≥ 9 items). 

In addition, the intervention exposure rate of the included studies was also extracted because this was suggested as an important quality criterion of ICT-based interventions [39,40]. The present review also assessed whether the included studies measured exposure frequency (ie, how frequently the participants accessed materials via ICT) of the intervention. For those studies that involved an Internet program, exposure duration (ie, how much time did participants spend on reading materials via ICT each time?) was also extracted [40].

## Results

### Selection of Articles

**Figure 1:**
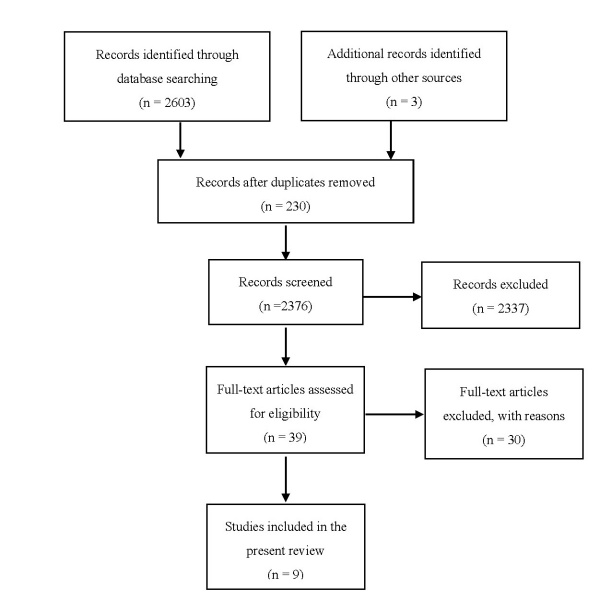
Search and selection process for the articles

The search and selection process for articles is illustrated in Figure 1. A total of 2606 articles were identified initially. After removing duplicates articles (n = 230) and irrelevant studies (n = 2337), 39 articles were retained for further considerations. Of these, 30 articles were excluded as they were descriptive or feasibility studies (n = 13), they were not targeted to the population of interest (n = 7), they did not use an RCT design (n = 6), they did not include any PA behavior outcome (n=3), or they did not use ICT as the mode of delivery (n = 1). In the end, 9 studies [41-49] were included in the present review.

### Data Extraction

Details regarding the 9 included studies are summarized in Table 1. All studies were published after 2000 and conducted in Western countries. Specifically, 6 studies were conducted in the United States [41,44,46-49], and 1 each was conducted in the United Kingdom [42], Australia [43], and New Zealand [45].

### Study Characteristics

In all, 8 studies [41,43-49] randomized the participants on an individual basis, while one study [42] randomized at the school level. Another 7 studies [41-45,48,49] delivered the intervention through ICTs in a home setting, whereas the remaining studies disseminated the intervention at schools [47] or clinics [46]. In regard to the target behavior, 3 studies [42,44,45] focused on PA behavior alone; 6 studies [41,43,46-49] focused on PA together with other health behaviors (eg, sedentary behavior, dietary intake, and diabetes management) (See Table 1).

### Participant Characteristics

The 9 included studies contained 1456 total participants and the sample sizes ranged from 57 to 473 in each study. Approximately 48.1% of the total sample participants were female (701/1456). In all, 6 studies included both genders [41,43,45-48], while 3 studies focused on a single gender: 2 studies [44,49] focused on females and 1 study focused on [42] males. In the majority of studies, the intervention was offered to healthy children and adolescents recruited from schools and communities (eg, a scout troop). Also, 3 studies focused on overweight [49] or diabetic patients [41,45] from pediatric clinics (See Table 1). 

### Intervention Characteristics

Intervention duration ranged from 2 weeks to 2 years. Of the 9 studies, 5 [42,44,45,47,48] focused on short-term results (≤ 3 months), 2 [43,46] focused on medium-term results (4-6 months), and 2 focused on long-term results (> 6 months) [41,49]. Regarding the mode of delivery, 8 studies [41-48] employed a single ICT mode (Internet = 4, email = 1, and SMS = 3) to deliver the intervention. The only exception was Williamson et al [49] who combined the use of Internet and email. The contacts made via the ICT mode varied from twice per day to once in 12 weeks. Also employed by 6 studies [41-43,45,46,48] were non-ICT modes (face-to-face, mail, and telephone) to contact the participants; the frequency of contact by non-ICT modes ranged from once per week to once every 3 months.

Of the 9 studies, 5 [41-43,46,47] had developed the intervention based on health behavior change theories: 3 [41-43] were guided by social cognitive theory (SCT) [50,51] and 2 [46,47] were developed based on SCT [50,51], transtheoretical model (TTM) [52], and relapse prevention model (RPM) [53]. Use of BCT in each study is summarized in Table 2. It was found that 3 types of BCT were used the most among the included studies: prompt specific goal setting, prompt self-monitoring of behavior, and provide feedback on performance. Overall, there was great variability in the number of BCTs used (the range was 3 to 9). In terms of the ICT initiation strategy, 5 studies [41,43,45-47] used investigator-initiated strategies and 4 studies [42,44,48,49] employed participant-initiated strategies.

**Table 1:** Overview of characteristics and main findings of included studies

Author, Year, and Country	Participant Characteristics	Study characteristics	Intervention Descriptions	Intervention Characteristics	Main Findings
Franklin et al, 2006, United States [41]	92 diabetic patients (8-18 years of age), 43 were female	Design: RCT Setting: home Focus: diabetes	ICT group 1: Sweet Talk SMS (automatic scheduled SMS reminder of the goal set, daily tips, and monthly text newsletter on diabetes issues. Participants could reply to the SMS and get an extra SMS for reply) plus intensive insulin treatment and standard treatment (clinic visit once per 3 to 4 months and access to emergency hotline). ICT group 2: Sweet Talk SMS in addition to conventional insulin treatment and standard treatment Control: standard treatment only	Duration: 1 year Mode and contact: 1 or 2 SMS per day^a^; 1 face-to-face every 3 to 4 months Theory: SCT Number of BCTs used: 8 Communication initiation: investigator	Significantly greater increase in perceived social support to exercise in both ICT groups compared with control group No significant difference between the 2 ICT groups
Jago et al, 2006, United Kingdom [42]	473 boy scouts (10-14 years), 0 were female	Design: cluster RCT Setting: home Focus: PA	ICT group 1: Internet-based PA program contained goal setting and a comic story on overcoming PA barriers plus face-to-face troop training started in the spring. ICT group 2: Internet-based PA program contained goal setting and a comic story on overcoming PA barriers plus face-to-face troop training started in the fall Control: dietary intervention plus face-to-face troop training	Duration: 9 weeks Mode and contact: 2 per week via Internet and 1 per week face-to-face Theory: SCT^a^ Number of BCTs used: 8 Communication initiation: participant	Significant increase in light PA in ICT group 1 No significant between-group differences
Lubans et al, 2009, Australia [43]	124 school children (mean age 14.1), 71 were female	Design: RCT Setting: home Focus: PA and Diet	ICT: social support email from investigator and pedometer self-monitoring plus printed PA and nutrition handbook and printed monthly newsletter for parents plus face-to-face school-based sport education program (structured PA session focusing on lifetime activities) plus information session (weekly diet and PA messages with teacher-demonstrated related activities to reinforce the message and summary lectures) Control: face-to-face school-based sport education program plus exercise handbook	Duration: 6 months Mode and contact: 1 email per week^a^, face-to-face contact once per week for the first 10 weeks Theory: SCT Number of BCTs used: 7 Communication initiation: investigator	Significantly greater increase in step counts in ICT group compared with control for both boys and girls Significantly greater increase in low active participants of ICT group compared with control group No significant difference in step counts in active participants between ICT group and control group
Marks et al, 2006, United States [44]	319 girls (12-14 years of age), that is, all 319 female	Design: RCT Setting: home Focus: PA	ICT: Web-based PA program containing interactive games, quiz, downloadable charts to plan daily activity, and audio demonstration of PA activities Control: print-based PA program with the content identical to the Internet-based intervention	Duration: 2 weeks Mode and contacts: Internet 2 times per week Theory: none mentioned Number of BCTs used: 3 Communication initiation: participant	Significant increase in PA self-efficacy and PA intention in both ICT and control groups Significantly greater increase in PA intention in control group compared with ICT group but not in PA, self-efficacy and PA level

Newton et al, 2009, New Zealand [45]	78 diabetic patients (11-18 years), 42 were female	Design: RCT Setting: home Focus: PA	ICT: pedometer self-monitoring with a goal of at least 10,000 step/day plus motivational SMS reminder (participants could reply the SMS and get an extra SMS for reply^a^) plus standard diabetes treatment Control: standard diabetes treatment	Duration: 12 weeks Mode and contact: 1 or 2 SMS contacts per week Theory: none mentioned Number of BCTs used: 5 Communication initiation: investigator	Both groups decreased in step counts, but this was not statistically significant. No significant between-group differences
Patrick et al, 2001, United States [46]	117 adolescents (11-18 years, mean age 14.1), 43 were female	Design: RCT Setting: clinic Focus: PA plus diet	ICT: interactive computer program (assessed and compared participant's self-reported PA and diet behavior with recommendations, gave feedback, and instructed the participants to select two behaviors that they are most ready to change and construct an action plan) plus provider counseling (provider review and discussion of the action with the participants) plus extended follow-up by mail for group 1 and by infrequent telephone plus mail contact for group 2, and frequent telephone plus mail contact for group 3 Control: interactive computer program plus provider counseling but no further extended follow-up	Duration: 16 weeks Mode and contact: Internet, face-to-face and mail, each once during the 16 weeks plus telephone once every 2 weeks Theories: SCT, TTM, and RPM Number of BCTs used: 8 Communication initiation: investigator	Significant improvement in moderate PA in all groups but no effect in vigorous PA No significant between-group differences
Prochaska et al, 2004, United States [47]	138 school children (12-14 years), 90 were female	Design: RCT Setting: school Focus: PA plus diet	ICT group 1: one-session Internet-based PA assessment with tailored feedback ICT group 2: one-session Internet-based PA plus dietary assessment with tailored feedback Control: no treatment	Duration: 12 weeks Mode and contact: 1 Internet contact in the 12 week period Theories: SCT, TTM, and RPM Number of BCTs used: 9 Communication initiation: investigator	Significantly greater increase in PA level in ICT groups compared with control groups for boys but not for girls

Shapiro et al, 2008, United States [48]	58 children (5-13 years), 36 were female	Design: RCT Setting: home Focus: PA plus diet plus sedentary activity	ICT: parent and child to report their sugar-sweetened beverages, screen time, and PA goals by SMS plus immediate and automatic SMS feedback plus 3 face-to-face psychologist-led educational sessions Control group 1: parent and child to report their sugar-sweetened beverages, screen time, and PA goals by using a paper diary plus 3 face-to-face psychologist-led educational sessions plus verbal feedback during the educational session Control group 2: 3 face-to-face psychologist-led educational sessions only	Duration: 8 weeks Mode and contact: 2 SMS per day, face-to-face once a week Theory: none mentioned Number of BCTs used: 7 Communication initiation: participants	No significant difference in step counts in all groups
Williamson et al, 2006, United States [49]	57 overweight girls (11-15 years), that is all 57 were female	Design: RCT Setting: home Focus: weight loss	ICT: Internet-based PA and dietary program with tailored information and prescriptions and online counseling plus 4 face-to-face meetings at 1, 3, 6, and 12 weeks Control: Internet-based program with general PA and dietary information plus 4 face-to-face sessions at 1, 3, 6, and12 months	Duration: 2 years Mode and contact: Internet once per week, email once per week, and face-to-face 4 times in 12 weeks Theory: none mentioned Number of BCTs used: 7 Communication initiation: participant	Significant improvement in self-reported exercise behavior in all groups No significant between-group differences

^a^ Information obtained from original author

**Table 2:** Use of behavior change techniques (indicated by Abraham and Michie [37])

Items	Franklin et al [41]	Jago et al [42]	Lubans et al [43]	Marks et al [44]	Newton et al [45]	Patrick et al [46]	Prochaska et al [47]	Shapiro et al [48]	Williamson et al [49]
Provide information about behavior-health link	X					X	X		X
Provide information on consequences				X		X	X		X
Provide information about other’s approval									
Prompt intention formation						X	X		
Prompt barrier identification		X	X			X	X		X
Provide general encouragement								X	
Set graded tasks		X							
Provide instruction	X	X	X						
Model or demonstrate the behavior		X	X	X					
Prompt specific goal setting	X	X			X	X	X	X	X
Prompt review of behavioral goals		X			X	X	X	X	
Prompt self-monitoring of behavior	X	X	X	X				X	X
Provide feedback on performance			X		X	X	X	X	X
Provide contingent rewards	X	X			X^a^			X	
Teach to use prompts and cues									
Agree on behavioral contract	X		X						X
Prompt practice	X				X			X	
Use follow-up prompts									
Provide opportunities for social comparison									
Plan social support or social change	X		X			X	X		
Prompt identification as a role model.									
Prompt self-talk									
Relapse prevention									
Stress management									
Motivational interviewing									
Time management									

^a^ Information obtained from original author

### Intervention Efficacy

Table 3 illustrates the effects of ICT-based interventions on PA behavior outcomes. Changes in behavioral variables were reported in 7 studies [42-48], and 4 of these [42,43,46,47] demonstrated significant within-group differences. Changes in psychosocial variables were presented in 3 studies [41,44,49], and all demonstrated significant within-group differences.

In all, 7 studies [41-45,47,48] compared the effects between an ICT group and a non-ICT control group. Of these, 3 [41,43,47] reported a positive effect favoring the ICT group, and 1 study showed a positive effect favoring the non-ICT control group [44]. In addition, 2 studies [46,49] contrasted the effect between two ICT groups. They examined whether different tailoring levels and follow-up methods would affect intervention efficacy. There were no significant between-group differences. On average, ICT-based interventions had a small effect size (0.03 to 0.41) on PA behavior change when compared with the control group. Notable exceptions were studies by Franklin et al [41], Lubans et al [43], and Prochaska et al [47], who reported large effect sizes.

### Assessment of Methodological Quality

In Table 4, the results of the methodological quality assessment are described. Of the 9 included studies, 7 [41-44,47-49] were rated as having good methodological quality. The low methodological quality scores were attributed to failure to report the concealment method for randomization, blinding of the assessors, and failure to follow-up long term. In all, 7 studies [41-44,46,47,49] measured intervention exposure frequency, while 5 studies [42,44,46,47,49] utilized an Internet program, and 3 of those studies [44,46,47] recorded Internet exposure duration.

**Table 3:** Effect of ICT-based intervention on PA outcomes

Sources		Effect^a^		
	Outcome Measure	Within-Group	Between-Group	Effect Size
Franklin et al [41]	Perceived social support to exercise	↑	+	0.76
Jago et al [42]	Light PA	↑	○	0.03
	Moderate PA	→	○	0.08
	Step counts	→	○	0.24
Lubans et al [43]	Step count, boys	↑	+	0.80
	Step count, girls	↑	+	1.2
Marks et al [44]	PA self-efficacy	↑	○	Not applicable
	PA intention	↑	—	0.41
	Self-reported PA	→	○	0.39
Newton et al [45]	Step counts	→	○	Not applicable
Patrick et al [46]	Moderate PA	↑	○	Not applicable
	Vigorous PA	→	○	Not applicable
Prochaska et al [47]	PA level, boys	↑	+	0.95
	PA level, girls	→	○	0.03
Shapiro et al. [48]	Self-reported PA	→	○	0.14
Williamson et al [49]	Self-reported exercise behavior	↑	○	Not applicable

^a^ The pre-post difference in PA behavior outcome in the intervention group was indicated by: “↑” for positive and significant, “→” for no significant change and “↓” for significant negative change. The pre-post difference in PA behavior outcome between the intervention group and control group was coded as “+” (significant difference favoring the ICT intervention group), “O” (no significant difference between groups), and “—” (significant difference favoring the control group).

**Table 4:** Assessment of methodological quality and intervention exposures of the studies

Items		Franklin et al [41]	Jago et al [42]	Lubans et al [43]	Marks et al [44]	Newton et al [45]	Patrick et al [46]	Prochaska et al [47]	Shapiro et al [48]	Williamson et al [49]
**Methodological quality**										
	Were the eligible criteria specified?	Yes	Yes	Yes	Yes	Yes	Yes	Yes	Yes	Yes
	Was the method of randomization described?	Yes	No	Yes	Yes	No	No	No	Yes	Yes
	Was the random allocation concealed? (ie, was the assignment generated by an independent person not responsible for determining eligibility of the participants)	Yes	Yes^a^	Yes	Unknown	Yes^a^	No^a^	Yes^a^	Yes^a^	No^a^
	Were the groups similar at baseline regarding important prognostic indicators?	Yes	Yes^a^	Yes	Yes	No^a^	Yes	Yes	Yes	Yes
	Were both the index and the control interventions explicitly described?	Yes	Yes	Yes	Yes	Yes	Yes	Yes	Yes	Yes
	Was the compliance or adherence with the interventions described?	Yes	Yes	No	Yes	Yes	Yes	Yes	Yes	Yes
	Was the outcome assessor blinded to the interventions?	No^a^	No^a^	No^a^	Unknown	No	Yes^a^	Yes^a^	No^a^	No^a^
	Was the dropout rate described, and were the characteristics of the dropouts compared with the completers of the study?	Yes	Yes	No	Yes	No	Yes	Yes	Yes	Yes
	Was a long-term follow-up measurement in both groups comparable?	Yes	No	Yes	No	No	No	No	No	Yes
	Was the timing of the outcome measurements in both groups comparable?	Yes	Yes	Yes	Yes	Yes	Yes	Yes	Yes	Yes
	Was the sample size for each group described by means of a power calculation?	Yes	Yes^a^	Yes	Yes	Yes	No^a^	Yes	No	Yes
	Did the analysis include an intention-to-treat analysis?	Yes	No	No^a^	Yes	Yes	No	Yes	No	Yes
	Were point estimates and measures of variability presented for the primary outcome measures?	Yes	Yes	Yes	Yes	Yes	Yes	Yes	Yes	Yes
**Intervention exposure**										
	Was the exposure frequency measured?	Yes^a^	Yes^a^	Yes	Yes	No	Yes	Yes	No	Yes
	Was the exposure duration measured?	Na	No	Na	Yes	Na	Yes^a^	Yes	Na	No

^a^ Information obtained from original author

Na, Not applicable

## Discussion

### Effects of ICT-based PA interventions for children and adolescents

The present review systematically evaluated the efficacy and methodological quality of ICT-based interventions that applied Internet and/or SMS as a delivery mode for PA behavior change in children and adolescents based on the evidence of randomized controlled trials during the past 12 years (1997-2009). As mentioned earlier, the review by Norman et al [33] only included 3 RCTs focused on children and adolescents. Although the present review located 6 more studies, the small number of included studies in both reviews indicates the needs for additional studies. The 3 RCTs reviewed by Norman et al were Internet-based interventions. In contrast, the present review illustrated that the proportion of Internet- and SMS-based interventions was almost equal. This finding suggests the emerging role of SMS in changing PA behaviors in a young population. However, there are no existing criteria to inform the choice of ICT for different research purposes. Future studies investigating this issue are suggested.

The present review demonstrates consistent evidence supporting the efficacy of improving psychosocial variables through ICT-based Interventions (eg, self-efficacy). For behavioral variables (ie, PA level), evidence was less consistent. Unfortunately, there is insufficient information explaining the underlying mechanisms for change because many of the included studies have an incomplete theoretical foundation. Baranowski and Jago [54] indicated that a complete theoretical foundation of an intervention played an imperative role in explaining the effects of PA interventions. Their framework stated that a complete theoretical foundation not only includes employing theory and theory-based strategies to design the intervention, but also an evaluation of mediating variables. Further, changes as a result of an intervention should be associated with changes in outcome variables and potential confounders (eg, gender and ethnicity) should also be assessed for their role in influencing the relationship between the mediating variables and the target behavior.

In the present review, only half of the studies reviewed developed their interventions based on health behavior change theories. We found that 4 theory-based interventions measured a behavioral variable (PA level) as the outcome and that only 2 studies [43,46] explicitly described how the desired outcomes were manipulated by the intervention components. None have measured changes in the theoretical constructs as the outcome variables. Although a few studies [42,43,47] analyzed the confounders (ie, season, baseline PA level, and gender) that serve as moderators for intervention effects, it was still difficult to determine the underlying mechanism that drives an intervention’s success and failure [55,56]. These findings reinforce the need to strengthen theoretical foundations in future studies.

In all, 7 studies [41-45,47,48] compared the effects of the ICT groups with either non-ICT or no treatment control groups. Also, 6 studies showed that ICT groups were either as effective as (n = 3) [42,45,48] or superior to (n = 3) [41,43,47] non-ICT groups. However, it is inconclusive whether ICT is equivalent or superior to other delivery approaches (ie, face-to-face). The problem with existing research is that the majority of the studies employed both ICT and face-to-face modes. In addition, these studies did not include a comparable control group. When analyzing the intervention characteristics of the ICT group and the non-ICT control group, the contact frequency of the two groups varied. For instance, Fanklin et al [41] and Lubans et al [43] employed ICT to provide tailored feedback to participants in the ICT group, but it was not offered to the non-ICT control group. Shapiro et al [48] gave tailored feedback to participants in both the ICT group and non-ICT control group, but the contact frequency in the ICT group (once per day) was far more frequent than in the non-ICT control group (once per week). Despite the fact that the impact of varied contact frequency on intervention efficacy was unclear, existing evidence [36] supported the notion that higher contact frequency was associated with enhanced efficacy. Although these studies reported significant between-group differences favoring the ICT group, it is difficult to determine whether the surplus effects in the ICT group were a result of the use of ICT or increased contact frequency. Nonetheless, the findings provide evidence supporting the effectiveness of ICT in PA interventions for children and adolescents, especially when used along with other delivery approaches.

In all, 3 studies [41,43,47] demonstrated significant between-group differences and large effect sizes, which are obviously larger than in the remaining studies. Attempts were made to examine whether any specific intervention characteristics contributed to larger effect sizes. We found that ICT-based interventions that were grounded in behavior change theory and utilized an investigator-initiation strategy were more likely to show more significant between-group differences and larger effect sizes than those did not (See Table 1).

There is extensive prior evidence suggesting that use of theory has a beneficial effect on interventions [26,55-57]. The mechanism that explained this evidence was that behavior change theories could inform researchers of the most influential mediating variables of the target behavior [58]. Through intervening on these influential mediating variables, people would be more likely to initiate behavior change. In this review, efforts were made to examine whether improved efficacy was associated with the use of a specific behavior change theory. Due to the small number of theory-based interventions and the heterogeneity of study designs, direct comparisons examining the effects across different behavior change theories on intervention efficacy could not be performed. Again, this finding indicates the importance of using a theoretical framework to facilitate an intervention’s success and designing experimental studies that compare the effects of different behavior change theories on intervention efficacy.

It is important to note that ICT-based interventions that used behavior change theory along with the adoption of investigator-initiated strategies showed significant between-group difference and larger effect size when compared with a non-ICT control group. A possible reason for the improved efficacy is that the investigator-initiation strategy uses a “pushed” approach [59], where automatic and specific materials (ie, Web hyperlinks and personalized feedback) are directly addressed to participant’s personal email or SMS. This can save participants the cognitive effort it takes to plan when, where, what, and how to prevent time conflicts with other daily tasks before using ICT to access the materials. All the participants have to do is to check their email and use their mobile phone in a typical fashion. This makes ICT-based interventions more compatible with a participant’s existing practice and lifestyle. According to the diffusion of innovation theory [60], increased compatibility of an intervention could enhance the likelihood that children and adolescents would read the materials and adhere to the interventions. Consequently, the initiation of behavior change could be more likely to happen [61]. However, the present review could not confirm the effect of ICT initiation strategy on intervention exposure and adherence rate since these data were not available in most of the included studies. Clearly, more studies are needed to investigate the impact of ICT initiation strategy on intervention exposure rate, adherence rate, and efficacy.

With respect to methodological quality, two-thirds of the included studies were classified as having good methodological quality. Most of the studies failed to report the allocation concealment and blinding of outcome assessments. Appropriate allocation concealment is important to avoid selection and confounding bias [62,63] while blinding of outcome assessment can lower the risk of exaggerating treatment effectiveness [64]. Absence of the above information will prevent us from appraising the risk of bias. Without understanding the risk of bias, we should remain cautious about the positive effects of ICT-based interventions [62,65]. Another shortcoming to this body of research is that very few studies (n = 2) conducted long-term follow up. As the development of a PA habit is a life-long task and maintenance of a new adopted behavior may require at least 6 months [66], interventions with a long-term follow-up period (> 6 months) may better allow us to assess the effects of ICT-based PA interventions.

There are two methodological issues related to the intervention exposure rate. First, there was improper reporting of the intervention exposure rate. In the present review, only 2 studies reported both exposure frequency and exposure duration. Second, there was an unclear description of the measurements used to assess intervention exposure rate. Williamson et al [49] was the only research group that employed objective measurements, and theirs was also the only study that defined intervention exposure rate and described how to measure it. Without an objective measure, the risk of response bias may increase [67]. These methodological issues have prevented us from estimating the extent to which the prescribed intervention dosage was received by the participants. If the dosage received by participants could not be estimated, it is hard to determine whether improvements in the measured outcomes were an effect of the interventions or other factors. Using Lubans et al [43] as an example, participant’s exposure to the social support email was low, but they still observed significant and positive results. It is possible that the positive effects were influenced by the face-to-face sports program. In addition, Crutzen and colleagues [68] suggested that intervention exposure rate may reflect the salience of intention of behavior change, which varied during different time periods throughout the intervention. It is important for researchers to understand participants’ behavior change patterns so necessary adjustments can be made. These findings indicate the need for adopting objective and valid instruments to measure intervention exposure rates. It is also important for future studies to report these data when presenting results.

### Conclusion

The present review provides evidence supporting the positive effects of ICTs in PA interventions for children and adolescents, especially when used with other delivery approaches (ie, face-to-face). Because ICT delivery approaches are often mixed with other approaches and these studies sometimes lack a comparable control group, additional research is needed to establish the true independent effects of ICT as an intervention delivery mode. Nevertheless, this review has found that combining the use of behavior change theory and investigator-initiated strategies could be associated with enhanced intervention efficacy and larger effect sizes. However, more studies are needed prior to reaching solid conclusions. Although two-thirds of the studies demonstrated satisfactory methodological quality, several quality criteria still have room for improvement (eg, providing clear descriptions of allocation concealment, blinding of outcome assessment, and intervention exposure rate). Furthermore, researchers should also consider intervening for a longer duration and employing objective instruments for assessing intervention exposure rate. Due to the small number of studies that met the inclusion criteria, researchers should be cautious when interpreting the findings of the present review.

## Abbreviations

BCT: behavior change technique
ICT: information and communication technology
PA: physical activity
RCT: randomized controlled trial
RPM: relapse prevention model
SCT: social cognitive theory
SMS: short message services
TTM: transtheoretical model
